# Evolutionary medicine: Why does prevalence of myopia significantly increase?

**DOI:** 10.1093/emph/eoy017

**Published:** 2018-06-28

**Authors:** Erping Long

**Affiliations:** Department of Ecology and Evolutionary Biology, University of Michigan, Ann Arbor, MI, USA

## DESCRIPTION OF THE CONDITION: MYOPIA

Myopia is characterized by the axial elongation of the eyeball which causes the image of distant objects to fall in front of the retina so that it cannot be brought into focus. Individuals with myopia experience blurred distant vision. Notably, high myopia significantly increases the risk of pathological ocular changes including retinal detachment, glaucoma, and myopic macular degeneration [1]. Myopia prevalence has approximately doubled in the past three decades, and it is estimated that 49.8% of the world population will develop the condition with 9.8% having severe myopia by the year 2050 [2].

**Figure 1.**
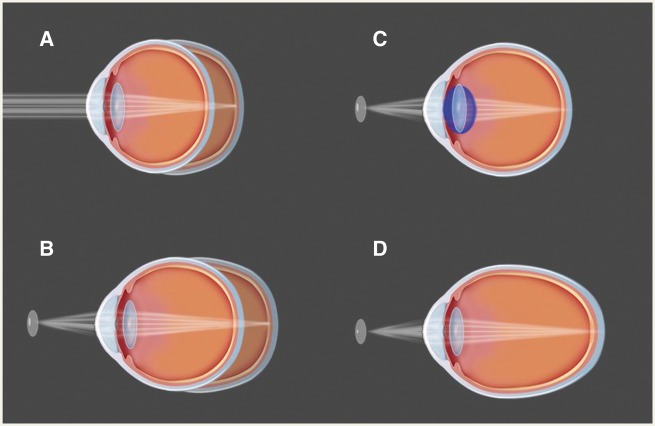
Emmetropization, myopia, and accommodation. **(A)** Human infants are born hyperopic, and then later on, their eyes exhibit gradual development relying on the visual inputs, eventually reaching emmetropization. **(B)** When a closer object is viewed, the image is focused behind the retina. The axial elongation of the eyeball is triggered and myopia happens. **(C)** For emmetropic eye, a closer object should be focused under the help of accommodation by increasing the optical power of the lens. **(D)** For myopic eye, closer objects can be focused without any accommodations

Eye growth is regulated by a homeostatic control process. Human infants are born hyperopic with the eyes exhibiting gradual development from visual inputs, eventually reaching emmetropization (Fig. 1A). The majority of our human ancestors were probably slightly hypermetropic or emmetropic, and thus had clear distant vision in order to monitor environmental dangers [3].

## EVOLUTIONARY PERSPECTIVES

Evolutionary mismatch is a concept commonly proposed in the context of rising rates of myopia. Here, we consider three different aspects of mismatch theory. First, a rapid increase in the use of electronic devices and the requirements of close work has influenced myopia prevalence in the modern world. According to the ‘retina-defocus’ hypothesis, myopia is induced by the defocused signals of near objects (Fig. 1B) [4]. As a result, the people with myopia are able to manage close work with substantially less accommodation (Fig. 1C and D). Second, increases in myopia have been associated with increased body stature (e.g. height) [5]. Increased stature involves evolutionary mismatch through improved access to high-calorie food [6]. Under this ‘increased stature’ hypothesis, phenotypic plasticity may result in non-adaptive changes to the length of the eye, producing myopia. Last, myopia has been proposed to relate to increasing time spent indoors in industrialized societies. Some evidence suggests that children who spend more time outdoors are less likely to become myopic, irrespective of how much near work they do [7]. Under this ‘indoor work’ hypothesis, the mechanism may involve light-stimulated release of dopamine, as increased dopamine can inhibit axial elongation [8].

## FUTURE IMPLICATIONS

The current strategies for controlling myopia, including low-dose atropine [9] and orthokeratology lenses [10], are effective but often confer side effects, and an urgent need exists for more concentration on the prevention and treatment of high myopia. Our evolutionarily perspectives provide further insight and justification for health policy-making to control myopia, including recommendations for children to spend more time outdoors during the day or using fewer electronic devices. However, before making such recommendations, more research is needed to disentangle the effects of these and other potential drivers of myopia, especially given that they covary in industrialized populations.

**Conflict of interest:** None declared.
